# Novel ACE2 fusion protein with adapting activity against SARS-CoV-2 variants *in vitro*

**DOI:** 10.3389/fimmu.2023.1112505

**Published:** 2023-03-08

**Authors:** Latifa Zekri, Natalia Ruetalo, Mary Christie, Carolin Walker, Timo Manz, Hans-Georg Rammensee, Helmut R. Salih, Michael Schindler, Gundram Jung

**Affiliations:** ^1^ Department of Immunology, Institute for Cell Biology, Eberhard Karls Universität Tübingen, Tübingen, Germany; ^2^ German Cancer Research Center (DKFZ) Partner Site Tübingen, German Cancer Consortium (DKTK), Tübingen, Germany; ^3^ Clinical Collaboration Unit Translational Immunology, Department of Internal Medicine, University Hospital Tübingen, Tübingen, Germany; ^4^ Cluster of Excellence iFIT (EXC 2180) “Image-Guided and Functionally Instructed Tumor Therapies”, University of Tübingen, Tübingen, Germany; ^5^ Institute for Medical Virology and Epidemiology, University Hospital Tübingen, Tübingen, Germany; ^6^ School of Life and Environmental Sciences and School of Life of Medical Sciences, The University of Sydney, Sydney, NSW, Australia

**Keywords:** SARS-CoV-2 therapy, ACE-2, neutralizing antibodies, immune escape, fusion protein

## Abstract

Despite the successful development of vaccines and neutralizing antibodies to limit the spread of severe acute respiratory syndrome coronavirus 2 (SARS-CoV-2), emerging variants prolong the pandemic and emphasize the persistent need to develop effective antiviral treatment regimens. Recombinant antibodies directed to the original SARS-CoV-2 have been successfully used to treat established viral disease. However, emerging viral variants escape the recognition by those antibodies. Here we report the engineering of an optimized ACE2 fusion protein, designated ACE2-M, which comprises a human IgG1 Fc domain with abrogated Fc-receptor binding linked to a catalytically-inactive ACE2 extracellular domain that displays increased apparent affinity to the B.1 spike protein. The affinity and neutralization capacity of ACE2-M is unaffected or even enhanced by mutations present in the spike protein of viral variants. In contrast, a recombinant neutralizing reference antibody, as well as antibodies present in the sera of vaccinated individuals, lose activity against such variants. With its potential to resist viral immune escape ACE2-M appears to be particularly valuable in the context of pandemic preparedness towards newly emerging coronaviruses.

## 1 Introduction

In the past two years, the Coronavirus disease 2019 (COVID-19) pandemic has claimed several millions of lives worldwide and has caused enormous -and unprecedented- social and economic damage (1, 2). Fortunately -and unprecedented as well- efficient vaccines have been developed and administered to millions of individuals in less than two years, and currently it appears that vaccination has become the cornerstone for the control of the pandemic worldwide (1, 3).

In the face of this truly remarkable success, the development of reagents for the treatment of established viral infections remains challenging. A growing understanding and appropriate treatment of the hyper-inflammatory and -coagulatory states occurring in the course of moderate and severe disease resulted in a significant reduction in mortality rates in treated patients. In addition, reagents with direct antiviral activity have been developed. Such reagents can be divided into two classes, small molecules with antiviral activity and neutralizing antiviral antibodies. For the latter, the tools of modern recombinant antibody technology, i. e., phage display, and single-cell cloning, have been used to generate optimized monoclonal antibodies with potent neutralizing capacity, directed to the receptor-binding domain (RBD) of the viral spike protein (S-protein) that binds to the ACE2 receptor on target cells (4–8). Several of these reagents have received approval for use during the early stages of infection. As of today, however, their activity in more advanced stages has been limited. Indeed, antibody-dependent enhancement (ADE), e.g., by non-neutralizing antibodies binding to viral particles, was reported to promote their Fc-mediated uptake by cells carrying Fc-receptors (FcRs), such as alveolar macrophages (9, 10).

However, a major limitation for the therapeutic activity of antibodies are recent mutations in SARS-CoV-2 variants that not only confer enhanced affinity to ACE2 and thus increased infectivity but also prevent the binding of antibodies raised against the B.1 S-protein (11–13).

A recombinant antibody approved for treatment of limited disease, REGN 10933 (14) exemplifies this strikingly. It strongly binds to the RBD of the B.1 S-protein but fails to bind to the S-protein encoded by known variants of concern (VOCs), such as the Beta and Omicron variants. The latter escapes effective neutralization by five of seven mAbs approved for treatment of COVID-19 (15–17). At the same time, the S-protein of the Omicron variant gained affinity towards the ACE2 protein (18, 19), resulting in increased infectivity.

In principle, the conceptual weakness of neutralizing antibodies directed to the RBD domain of the S-protein discussed above might be overcome by recombinant Fc-based fusion proteins comprising the “natural” binding partner of the RBD domain, the ACE2 protein. In contrast to RBD binding antibodies, the neutralizing capacity of such proteins would not be impaired but rather strengthened by affinity gaining mutations in the RBD. Moreover, since the RBD ACE2 interaction is mediated by a dimeric form of ACE2, an Fc based format may promote ACE2 dimerization (20). Despite this conceptual advantage, the construction of such fusion proteins faces challenges as well: first, the affinity of recombinant ACE2 to viral S-proteins is lower than that of most antibodies. Second, the enzymatic activity of physiologically expressed ACE2 is critical for the proper function of the renin-angiotensin-aldosterone system (RAAS). This system is of vital importance, among others, for blood pressure regulation, and high doses of enzymatically active protein might induce uncontrollable side effects. Although it has been suggested that ACE2 may function as a “rescue protein” in the course of the SARS-CoV-2 infection (21), we share the view expressed in a paper Khodarahmi et al. (22), that recommend the use of enzymatically inactive ACE2 if blockade of the S-protein is intended.

Based on the considerations outlined above, we have constructed and characterized an ACE2-Fc fusion protein designated ACE2-M, that carries mutations to:

- abrogate FcR binding and complement activation by the Fc domain
- deplete enzymatic activity of the ACE2 protein
- enhance the apparent affinity of the ACE2 S-protein interaction

Here we evaluate the capability of ACE2-M to bind and to neutralize various virus variants. This activity was benchmarked against the therapeutic antibody REGN 10933 and the serum of vaccinated individuals.

## 2 Materials and methods

### 2.1 Generation and production of ACE2 fusion proteins and REGN 10933

The human ACE2 extracellular domain (aa 18-740; Gene ID: 59272) and the variable domain sequences of the REGN 10933 antibody (14) were codon-optimized for expression by Chinese hamster cells using the GeneArt GeneOptimizer tool (Thermo Fisher Scientific, Regensburg, Germany). VH, VL, and ACE2 sequences (ACE2 wild-type (ACE2) or the indicated mutants, ACE2-RR, ACE2-K, ACE2-M) were synthesized *de novo* at GeneArt (Thermo Fisher Scientific, Regensburg, Germany). ACE-2 coding sequences were fused at their C-terminus to a human Igγ1 Hinge- Fc domain *via* a flexible (GGGGS)^3^ linker. Modifications in the CH2 domain consisting of the amino acid substitutions and deletions E233P; L234V; L235A; ΔG236; D265G; A327Q; A330S (EU index), which abrogate FcR binding and complement fixation, were introduced as described (23). REGN 10933 variable sequences were inserted into a human Igγ1 backbone comprising CH1‐CH2‐CH3‐ or CΚ‐constant domain sequences as described (23). All constructs were transiently transfected and produced using the ExpiCHO™ Expression System (Thermo Fisher Scientific, Regensburg, Germany) according to the manufacturer’s instructions and were then purified by HiTrap™ MabSelect™ SuRe columns (Cytiva, Freiburg, Germany), before being subjected to preparative and analytical size exclusion chromatography (SEC) using HiLoad™ 16/600 Superdex 200 pg and Superdex™ 200 Increase 10/300 GL columns (Cytiva Freiburg, Germany), respectively. Endotoxin levels of samples, as determined by a limulus amebocyte lysate assay (Endosafe^®^Charles River, Charleston, SC), were < 0.5 EU/ml. Sodium dodecyl sulfate-polyacrylamide gel electrophoresis (SDS-PAGE) was performed as previously described (23).

### 2.2 Spike proteins

SARS-CoV-2 full-length trimeric spike proteins corresponding to the B.1 (B.1.126), Alpha (B.1.1.7), Beta (B1.351), Gamma (P.1), and Delta (B.1.617.2) were purchased from BioServ, (Sheffield, UK). The Trimeric S-protein corresponding to Omicron (B.1.1.529) or Omicron subvariant BA.5 and BQ1.1 were obtained from Sino Biological (Beijing, China).

### 2.3 ACE2 catalytic activity assay

Enzymatic activity of ACE2 fusion proteins was measured using the ACE2 Activity Assay Kit (Fluorometric) (BioVision, Milpitas, CA) according to the manufacturer’s instructions. The proteins were diluted in assay buffer to 22.7, 4.54, and 0.91 nM final concentration. Fluorescence was measured using a Wallac 1420 Victor 2 Multi-Label Counter (Perkin-Elmer, Waltham, MA).

### 2.4 Competitive ELISA

The indicated S trimeric proteins were coated on 96-well plates at 1µg/ml, 4°C overnight. After washing, wells were blocked with PBS containing 3% BSA for 1 hour at room temperature. Next, a serial dilution of the indicated ACE2 fusion proteins, REGN 10933 or serum antibodies were pre-mixed with 150 nM of His-tagged ACE2 wild-type protein (BioLegend, San Diego, CA) and added to the plates. In case of the Omicron variants (Sino Biological), the His-tagged ACE2 protein was additionally biotinylated using the One-Step Biotinylation Kit (Miltenyi, Cologne, Germany) according to the manufacturer’s instructions. For visualization, a Penta-His HRP conjugate (1:1000) (Qiagen, Hilden, Germany) or mouse anti-Biotin HRP conjugate (1:1000) (Invitrogen, Waltham, MA) were used. Unbound HRP-conjugated antibodies were removed by washing, TMB substrate was added, and absorbance was measured at 450nm.

### 2.5 Determination of anti-spike antibodies in the sera of vaccine recipients

Sera were collected from 8 healthy donors (25-65 years of age). All donors received a first dose of Vaxzevria vaccine and then a second dose of Comirnaty or Spikevax. Sera were collected 40-45 days after the second dose and anti-SARS-CoV-2 antibody concentrations were measured using the Euroimmun Anti-SARS CoV-2 ELISA IgG kit (Euroimmun, Luebeck, Germany). Briefly, serum samples were diluted at 1:100 and 1:1000 and ELISA was performed following manufacturer’s instructions. A titration of a reference neutralizing antibody (REGN 10933) was used to calibrate the assay.

### 2.6 Measurement of fusion- and spike protein interaction by biolayer interferometry

Trimeric S-proteins were analyzed for their binding to ACE2-RR or ACE2-M fusion proteins using an Octet HTX system (Sartorius, Goettingen, Germany). Assays were run with a sensor offset of 3 mm and an acquisition rate of 5 Hz on AHC biosensors in 16-channel mode. Microplates were loaded with 60 μL per well of assay buffer consisting of PBS with 0.05% Tween-20 and 0.1% BSA. Sensors were equilibrated in assay buffer for 10 min. Following a baseline step of 60s, the analyte S-proteins were loaded for 120 s. Association was measured for 150s and dissociation for 300s. Regeneration of the sensors was performed using 10 mM Glycine pH 1.5. Data evaluation was done using Octet Analysis HT Software. The reference subtraction was performed to consider the potential dissociation of analyte loaded onto the biosensor. Data traces were aligned to the baseline, followed by an inter-step correction for the dissociation step. Savitzky-Golay filtering was applied to the data and the curves were fitted globally using a 1:1 binding model (with Rmax unlinked by sensor).

### 2.7 Viruses

All experiments with SARS-CoV-2 viruses were conducted in a Biosafety Level 3 laboratory at the University Hospital Tübingen. The SARS-CoV-2 strain icSARS-CoV-2-mNG (24) was obtained from the World Center for Emerging Viruses and Arboviruses (WRCEVA) of the UTMB (University of Texas Medical Branch, Galveston, TX, USA). SARS-CoV-2 B.1.126 (parental D614G), referred as B.1, and SARS-CoV-2 B.1.351 (Beta), were isolated from patient samples and variant identity was confirmed by next-generation sequencing of the entire viral genome as described before (25, 26). SARS-CoV-2 B.1.1.529 (Omicron) was isolated from a throat swab collected in December 2021 at the Institute for Medical Virology and Epidemiology of Viral Diseases, University Hospital Tübingen, from a PCR-positive patient. Fifty microliters of patient material were diluted in medium and used directly to inoculate 150,000 Caco-2 cells in a six-well plate. 48 hours post-infection (hpi), the supernatant was collected, centrifuged, and stored at -80°C. After two consecutive passages, an RNA sample from the supernatant was prepared, and NGS confirmed that the clinical isolate belongs to the lineage B.1.1.529. All virus stocks were generated in Caco-2 cells collecting supernatants 48-72 hpi. Multiplicity of infection determination (MOI) was conducted by titration using serial dilutions of both virus stocks. The number of infectious virus particles per ml was calculated as (MOI × cell number)/(infection volume), where MOI = -ln (1-infection rate).

### 2.8 Virus neutralization assay

Caco-2 (Human Colorectal adenocarcinoma, ATCC HTB-37) cells were cultured at 37°C with 5% CO2 in DMEM containing 10% FCS, 2 mM L-glutamine, 100 mg/ml penicillin-streptomycin and 1% NEAA. Neutralization assays using clinical isolates were performed as described in Wagner et al., 2021. Briefly, cells were co-incubated with the clinical isolate SARS-CoV-2 B.1.126, SARS-CoV-2 B.1.351 (Beta), or SARS-CoV-2 B.1.1.529 (Omicron), at MOI of 0.7-4.0, and serial dilutions of the ACE2 protein designs. 48 hpi, cells were fixed with 80% acetone, and immunofluorescence (IF) staining was performed using an anti-SARS-CoV-2 nucleocapsid antibody (GeneTex, Cat No. GTX135357) and goat anti-rabbit Alexa594-conjugated secondary antibody. Cells were counterstained with DAPI solution and images were taken with the Cytation3 (BioTek). Infection rates were calculated as the ratio of Alexa594-positive over DAPI-positive cells, which were automatically counted by the Gen5 software (BioTek). Inhibitory concentration 50 (IC50) was calculated as the half-maximal inhibitory dose using four-parameter nonlinear regression (GraphPad Prism).

### 2.9 Binding of ACE2 fusion proteins to SARS-CoV-2 infected cells

For binding experiments, 3×10^6^ Caco-2 cells were seeded in a T75 flask the day before infection, in a medium containing 5% FCS. Cells were infected with SARS-CoV-2-mNG, and 48 hpi cells were detached from the flask using Accutase, fixed with 2% PFA for 10 minutes at 37°C, and resuspended in FACS buffer (PBS, 1%FCS). 1×10^6^ cells in 100 µL in FACS buffer were distributed in a U-shape 96-well plate. The plate was centrifuged at 600 g for 5 min and the buffer was removed by a fast decant. Cells were incubated for 1h at 4°C using 50 µl of 3-fold serial dilutions of ACE2 protein or REGN 10933, tested from 40 µg/ml following 12 dilution points. Cells were washed with 150 µl of FACS buffer/well, centrifuged, and the supernatant decanted. The washing step was repeated using 200 µl of FACS buffer/well. Subsequently, cells were incubated with 50 µl of a 1:200 dilution of the Secondary AB- R-Phycoerythrin (PE) conjugated affinity pure F(ab’)2 Fragment Goat-anti Human IgG-Fc gamma fragment (Jackson-Immuno) for 30 minutes at 4°C. The two washing steps were repeated, and the cells were resuspended using 100 µl of FACS buffer/well. Controls included: mock-infected cells incubated with the highest and lower protein concentrations; infected cells non-incubated as well as infected cells stained only with the secondary antibody. Alternatively, Caco-2 cells were infected with SARS-CoV-2 parental or Omicron variants and the same protocol described above was followed. After incubation with PE-secondary antibody, cells were permeabilized with 80% Acetone for 5 minutes at room temperature, washed as described before and immunofluorescence (IF) staining was performed using an anti-SARS-CoV-2 nucleocapsid antibody (GeneTex, Cat No. GTX135357) (1:1000, 1 h) and goat anti-rabbit Alexa594-conjugated secondary antibody (1:2000, 1 h). After the final washing steps, the cells were analyzed using a MACSQuant VYB (Miltenyi). FACS analysis was performed with MACS Quantify Software (Miltenyi) and Flowlogic (Miltenyi–Inivai).

## 3 Results and discussion

### 3.1 Construction and characterization of ACE2-M

ACE2 fusion proteins were constructed by fusing the ACE2 extracellular domain to a human IgG1 Fc fragment that was originally developed to prevent binding to FcRs by T cell activating bispecific antibodies. This modification consists of 6 mutations and one deletion in the CH2 domain of the human IgG1 Fc domain that completely abrogates binding to FcRs and ablates complement activation (23).

To avoid the introduction of undesired peptidase activity, two catalytically-inactive forms of ACE2 were generated. The first mutant, ACE2-RR, contains two substitutions in the catalytic pocket, H374R, and H378R, that prevent zinc binding within the active site of the protein (27). The second mutant, ACE2-K, has a single mutation at position 273 (R273K), which is critical for enzymatic activity (28). Fc-fusion proteins constructed with either of the two variants show the size expected for a dimeric molecule as demonstrated by SDS-PAGE and size exclusion chromatography (**Supplementary Figures 1A–C**). In contrast to ACE2-RR, ACE2-K retained some residual enzymatic activity and showed a slight loss in binding affinity to recombinant trimeric parental S-protein as measured by enzyme-linked immunosorbent assay (ELISA) (**Supplementary Figures 1D, E**). Thus, the ACE2-RR version of the fusion protein was selected for further modification.

**Figure 1:**
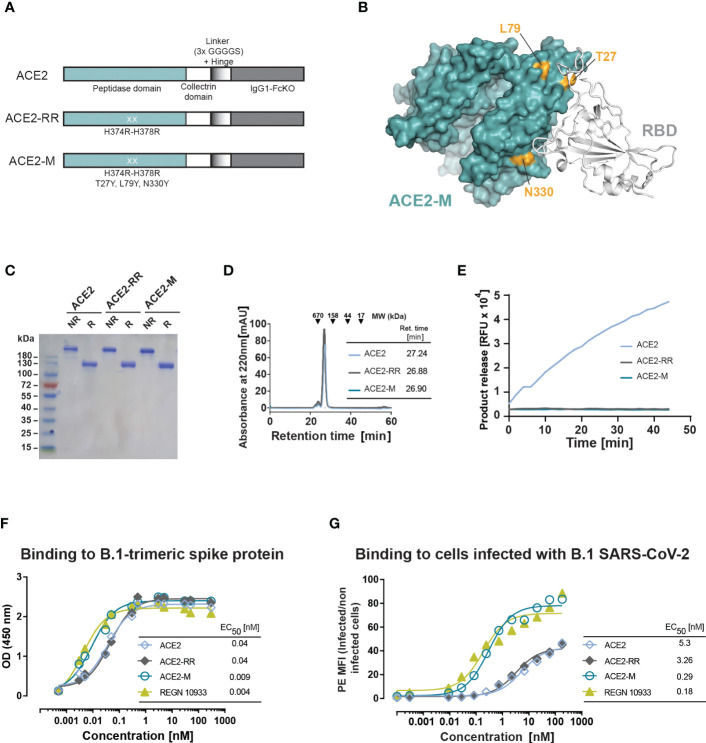
Design and biochemical characterization of ACE2-M. **(A)** Schematic representation of the ACE2 molecules used in this study. The ACE2 extracellular domain, comprising peptidase and collectrin domains, was fused to an IgG1-Hinge-Fc-Ko domain *via* a flexible linker (GGGGS)3 (ACE2 version). The H374R-H378R mutations were introduced to abolish ACE2 enzymatic activity (ACE2-RR version). Additional mutations were introduced to increase binding to the S-protein as indicated in **(A, B)**. **(B)** Structure of ACE2 in complex with S-RBD (using PDB 6M0J) with residues substituted in ACE2-M highlighted in gold. **(C)** Coomassie-stained gel of the three generated fusion proteins. NR, non-reduced; R, reduced. **(D)** Superposed analytic chromatography profiles of the ACE2 proteins run on a Superdex S200 Increase 10/300GL column. The table indicates the corresponding retention times. **(E)** Enzymatic activity of the ACE2 fusion proteins (4.5 nM) as measured by cleavage of a fluorescent peptide substrate. **(F)** Binding of the fusion proteins and REGN 10933 to B.1 trimeric S-protein determined by ELISA. Results represent the standard deviation (SD) of n=3. EC_50_ as calculated by the GraphPad software. **(G)** Binding of the fusion proteins and REGN 10933 to Caco-2 cells infected with the recombinant infectious clone SARS-CoV-2 that expresses the mNeonGreen as a reporter gene. Binding was assessed by flow cytometry.

To enhance binding to the S-protein, we introduced three additional mutations to the ACE2-RR-Fc fusion protein that are located at the S-protein binding surface of ACE2: T27Y, L79Y, and N330Y (**Figures 1A, B**). These mutations are similar to an ACE2 variant described by Chan and collaborators (29). ACE2-M, was produced as a stable dimer and had no detectable enzymatic activity (**Figures 1C–E**).

We next measured the binding affinity of recombinant trimeric S-protein and ACE2-M by ELISA, which was benchmarked against an Fc-fusion protein comprising the wild type ACE2 protein, as well as against the reference antibody REGN10933. In this assay, ACE2-M showed a 4-fold higher binding affinity than wild-type ACE2 fusion protein (**Figure 1F**). Interestingly, the apparent binding affinity measured by flow cytometry of Caco-2 cells infected with a recombinant parental virus resulted in a greater than 18-fold affinity enhancement (**Figure 1G**). In both assays, the S-protein apparent binding affinity of ACE2-M and REGN 10933 was similar. The differences between the affinities of antibodies and fusion proteins to S-proteins coated to an ELISA plate compared with those embedded in the membrane of a virus-infected cell are noteworthy. Obviously, binding to infected cells resembles the “physiological state” more closely, and may predict different neutralization capacities of the various fusion proteins more reliably as demonstrated below.

To test whether ACE2-M retains binding to the globally spreading SARS-CoV-2 VOCs Alpha (B.1.1.7), Beta (B1.351), Gamma (P.1), Delta (B.1.617.2), and Omicron (B.1.1.529), structural analysis of ACE2-M and the respective S-RBD variants was performed (**Supplementary Figure 2**). This analysis revealed that the affinity-enhancing mutations introduced into the ACE2-M protein appear to be spared by the viral escape mechanism, probably because they form part of the RBD-binding interface. Those observations were further confirmed by measuring the binding kinetics of ACE2-M to all variants by biolayer interferometry technology (BLI). The results depicted in **Supplementary Figure 3** demonstrate a superior apparent binding affinity of ACE2-M to spike variants. Again, the differences in affinities were less pronounced compared to those observed by flow cytometry of infected cells (**Figure 1G**).

### 3.2 ACE2-M resists viral escape, in contrast to REGN 10933

**Figure 2:**
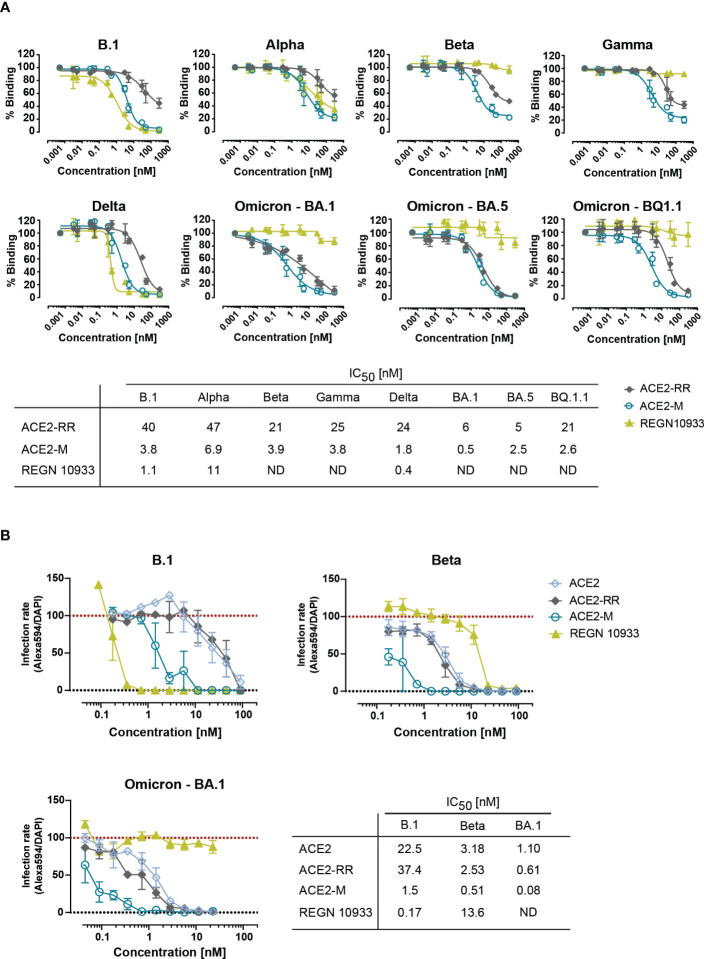
Competitive binding of fusion proteins and REGN 10933 to ACE2 and their capacity to neutralize parental and SARS-CoV-2 VOCs. **(A)** Competitive ELISA was performed by immobilizing the indicated full length S-proteins. The results depicted show the binding of 150 nM of His-tagged ACE2 wild-type protein, that was competed with the indicated concentrations of the ACE2 fusion molecules or REGN 10933 antibody. The error bars represent the SD of two independent experiments with technical replicates. The IC_50_ values summarized in the table were calculated using the GraphPad software. ND: Not detected. **(B)** Neutralization capacity was determined as described in material and methods. Briefly, Caco-2 cells were infected with the indicated clinical isolates of SARS-CoV-2. Infection rates, calculated as the number of infected cells (Alexa594+) over the total number of cells (DAPI+), were normalized to virus-only infection control. Mean and SEM values are calculated from three independent experiments with technical duplicates. Neutralization assay corresponding IC_50_ values were calculated using GraphPad software. ND, Not detectable.

Next, we tested the ability of ACE2-RR, ACE2-M, and REGN 10933 to inhibit binding of ACE2 to various S-proteins by competition ELISA. To this end, the fusion proteins and REGN 10933 were mixed with a saturating amount of His-tagged-ACE2 protein before binding to immobilized S-proteins was determined. In line with the BLI results, we observed an improved inhibition of binding by ACE2-M to all S-proteins (**Figure 2A**). In contrast, REGN 10933 failed to inhibit ACE2 binding to Beta, Gamma, and Omicron S-proteins. These results are consistent with previously published data, suggesting that the reduced binding of REGN 10933 to certain S variants is likely due to mutations in amino acids K417 and E484 of the S-protein (30, 31), found in the Beta, Gamma, and Omicron variants.

Likewise, binding of ACE2 fusion proteins and REGN 10933 antibody to Caco-2 cells infected with authentic SARS-CoV-2 natural isolates corresponding to the parental strain (D614G) and Omicron (B.1.1.529) showed similar results (**Supplementary Figure 4**). REGN 10933 bound with high affinity to Caco-2 cells infected with the parental virus, in line with the results obtained with the recombinant virus in **Figure 1G**. However, REGN 10933 failed to bind to Caco-2 cells infected with the Omicron virus. In contrast, ACE2-M retains high-affinity binding to Caco-2 cells infected with either isolate. Unexpectedly, we observed a 4-fold lower MFI for binding to cells infected with Omicron vs. parental virus. This observation could be explained by the reduced replication capacity recently described for Omicron variant (32–34). The reduced replication seems to be due to the inefficient use of the cellular protease TMPRSS2, which promotes cell entry through plasma membrane fusion (33). We speculate that reduced replication in Caco-2 cells may result in a decreased expression of S-proteins on the cell surface.

To investigate the efficacy of ACE2-M in preventing viral infection, we performed a neutralization assay in Caco-2 cells using SARS-CoV-2 natural isolates from the parental B.1 as well as Beta, and Omicron strains. ACE2-M neutralized all SARS-CoV-2 infected cells at picomolar concentrations (**Figure 2B** and **Supplementary Figure 5**). ACE2-M activity was greater against Omicron than against the Beta variant or the parental B.1. In contrast, the activity of REGN 10933 against the Beta and Omicron variants was reduced and undetectable, respectively. Altogether, our results demonstrate that binding of ACE2-M to S-variants (vs. parental) is preserved or enhanced (in case of Omicron), while it is weakened or lost in the case of REGN 10933. Thus, these results confirm our “founding hypothesis” for the construction of ACE2 fusion proteins, namely that viral variants will mutate “away” from recognition by antibodies but “towards” recognition by ACE2 fusion proteins and hence to neutralization by such proteins.

### 3.3 SARS-CoV-2 VOCs are able to evade vaccine-elicited antibodies but not ACE2-M

**Figure 3:**
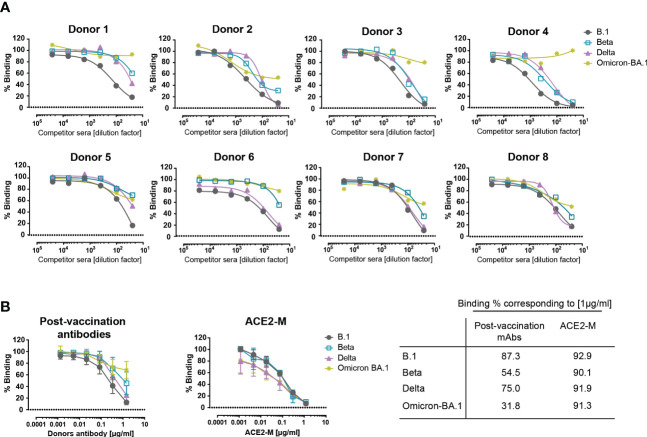
Competitive binding of antibodies in the sera of vaccinated donors and ACE2-M to various spike proteins. **(A)** Similar competition ELISA assay to the one described in **Figure 1A** was performed with sera from 8 healthy vaccinated individuals. Each serum sample was diluted in a range of 1/25 to 1/25600 and tested against His-tagged ACE2 for binding to SARS-CoV-2 B.1, Beta, Delta, and Omicron – BA.1 S-protein variants. **(B)** SARS-CoV-2 antibodies were quantified in donor sera using the Euroimmun ELISA. Equal amounts of antibody or ACE2-M were diluted in SARS-CoV-2 negative serum and tested in a competition ELISA for their ability to compete with wild-type ACE2-His for S-protein binding. The table summarizes the percentage binding achieved by a protein concentration of 1µg/ml.

To evaluate the binding efficiency of antibodies generated by active immunization to parental, Beta, Delta, and Omicron trimeric S-proteins, sera from 8 healthy fully vaccinated donors were used and evaluated in a competitive ELISA similar to that described above in **Figure 2A**. Similar to REGN 10933, antibodies present in the post-vaccination sera showed a reduction in binding to Beta, Delta, and, to a greater extent, to Omicron S-proteins (**Figure 3A**). These results suggest that antibodies generated after active immunization against the parental S-protein are less effective against new variants in accordance with recent reports (35–40).

Next, we defined the required amount of neutralizing antibodies and ACE2-M protein, to achieve a complete inhibition of binding to S-proteins. To this end, SARS-CoV-2 antibody concentrations present in the sera of vaccinated donors were quantified, adjusted, and compared in a competitive ELISA to the ACE2-M protein. Our results, depicted in **Figure 3B**, show that ACE2-M, at a concentration of 1 µg/ml, achieved almost complete binding inhibition of ACE2 wild type to all S-variants, however, only a partial binding inhibition could be obtained with antibodies in the various sera. Of note, a serum concentration of 1 µg/ml is easily reached during treatment of antiviral disease with suitable monoclonal antibodies (41, 42).

We have demonstrated that ACE2 fusion proteins are not subject to immune escape exerted by variants of the S-protein in line with recent publications (43–46). In fact, variants with increased affinity such as Omicron variants (BA.1, BA.5 and BQ1.1), are neutralized more effectively than the parental B.1. Recently, alternative receptors have been reported to interact with the SARS-CoV-2 virus (47–51). In cells with low ACE2 expression, it is thought that SARS-CoV-2 can enter the cells *via* several alternative receptors, but the entry mechanism remains to be defined. Although there is no direct evidence that SARS-CoV-2 escapes ACE2 treatment, the fact that the highly transmissible variant Omicron BA.1 has evolved to be less dependent on TMPRSS2, raises the possibility that alternative SARS-CoV-2 mutations may also contribute to viral evolution and may cause ACE2 immune escape.

To our knowledge, ACE2-M is the first engineered ACE2 fusion molecule combining modifications for Fc-attenuation, enzymatic depletion, and ACE2 affinity enhancement. Although animal studies are required to conclusively evaluate the importance of enzymatic depletion and Fc-attenuation, this molecule provides an important additional option for treatment of COVID-19 and other coronaviruses that use the ACE2 protein as entry receptor.

## Data availability statement

The original contributions presented in the study are included in the article/**Supplementary Material**. Further inquiries can be directed to the corresponding author.

## Ethics statement

The studies involving human participants were reviewed and approved by ethics committee of the Faculty of Medicine of the Eberhard Karls Universitaet Tuebingen and of the University Hospital Tuebingen. The patients/participants provided their written informed consent to participate in this study.

## Author contributions

GJ and LZ designed the study. LZ, NR, MS and GJ designed and interpreted the results. LZ produced and characterized ACE2 fusion proteins. MC designed the affinity maturated mutant and performed structural analysis. LZ and CW performed the *in vitro* binding and competition assays. NR performed the binding analysis on virus-infected cells and neutralization assays. TM and LZ conducted BLI analysis. HS and H-GR contributed to interpretation of the experimental results. LZ wrote the manuscript. GJ, MS, MC and NR critically revised the manuscript. All authors read and accepted the submitted manuscript.
