# Supplementary material for: Novel ACE2 fusion protein with adapting activity against SARS-CoV-2 variants in vitro
Source: Front Immunol. 2023 Mar 8;14:1112505. doi: 10.3389/fimmu.2023.1112505 (PMC10030959; doi:10.3389/fimmu.2023.1112505)
Supplement: Supplementary file 1 [file Presentation_1.pdf]

Supplementary Material

Novel ACE2 fusion protein with adapting activity against SARS-CoV-2 variants *in vitro*

Latifa Zekri[1,2,3,4*], Natalia Ruetalo[5], Mary Christie[6], Carolin Walker[1,2], Timo Manz[1,2], Hans-Georg Rammensee[1,2,4], Helmut R Salih[2,3,4], Michael Schindler[5†] and Gundram Jung[1,2,4†]

[1] Department of Immunology, Institute for Cell Biology, Eberhard Karls Universität Tübingen, Germany, Tübingen, Germany

[2] German Cancer Research Center (DKFZ), Partner Site Tübingen, German Cancer Consortium (DKTK), Tübingen, Germany

[3] Clinical Collaboration Unit Translational Immunology, German Cancer Consortium (DKTK), Department of Internal Medicine, University Hospital Tübingen, Tübingen, Germany

[4] Cluster of Excellence iFIT (EXC 2180) "Image-Guided and Functionally Instructed Tumor Therapies", University of Tübingen, Tübingen, Germany

[5] Institute for Medical Virology and Epidemiology, University Hospital Tübingen, Tübingen, Germany

[6] School of Life and Environmental Sciences and School of Life of Medical Sciences, The University of Sydney, NSW, Australia.

[†] Shared last authors

*** Correspondence:**
Latifa Zekri, Ph.D
Email: latifa.zekri@ifiz.uni-tuebingen.de

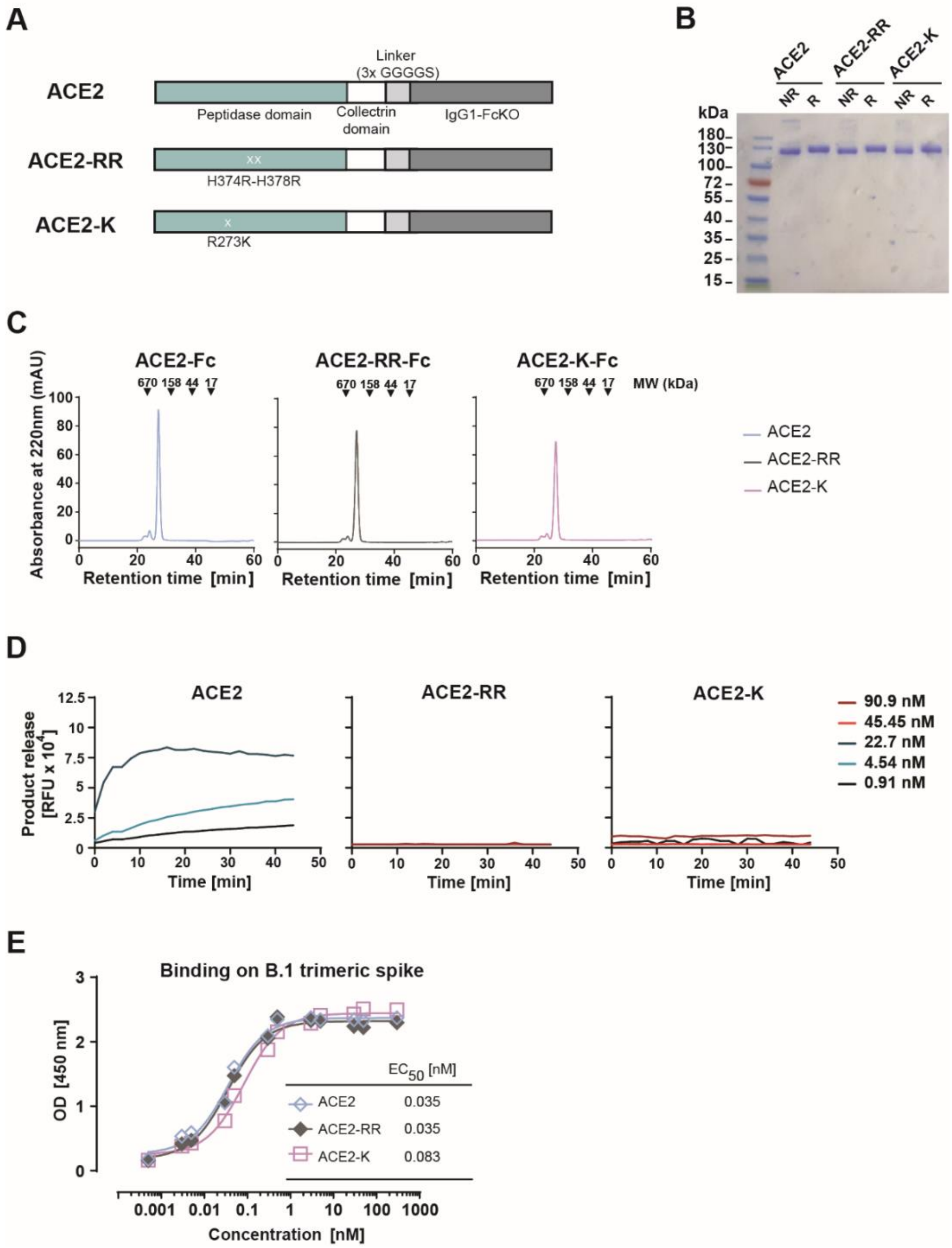

Supplementary Figure 1. Characterization of ACE2 molecules with defective enzymatic activity

(A) Schematic representation of the designed ACE2 molecules containing mutations in the peptidase domain that have been described to abolish the ACE2 enzymatic activity: ACE2-RR contains H374R-H378R and ACE2-K contains R273K. **(B)** SDS-PAGE gel of the generated fusion proteins. NR: non-reduced; R: reduced. **(C)** Analytical Size exclusion chromatography profiles of the ACE2 proteins using a Superdex 200 Increase 10/300GL column. **(D)** The enzymatic activity of ACE2 molecules was determined by measuring the fluorescent product from the cleavage of the peptide substrate. **(E)** Binding titration to S-trimeric protein by ELISA. The results represent the standard deviation (SD) of n=2. EC_{50} values were defined by GraphPad.

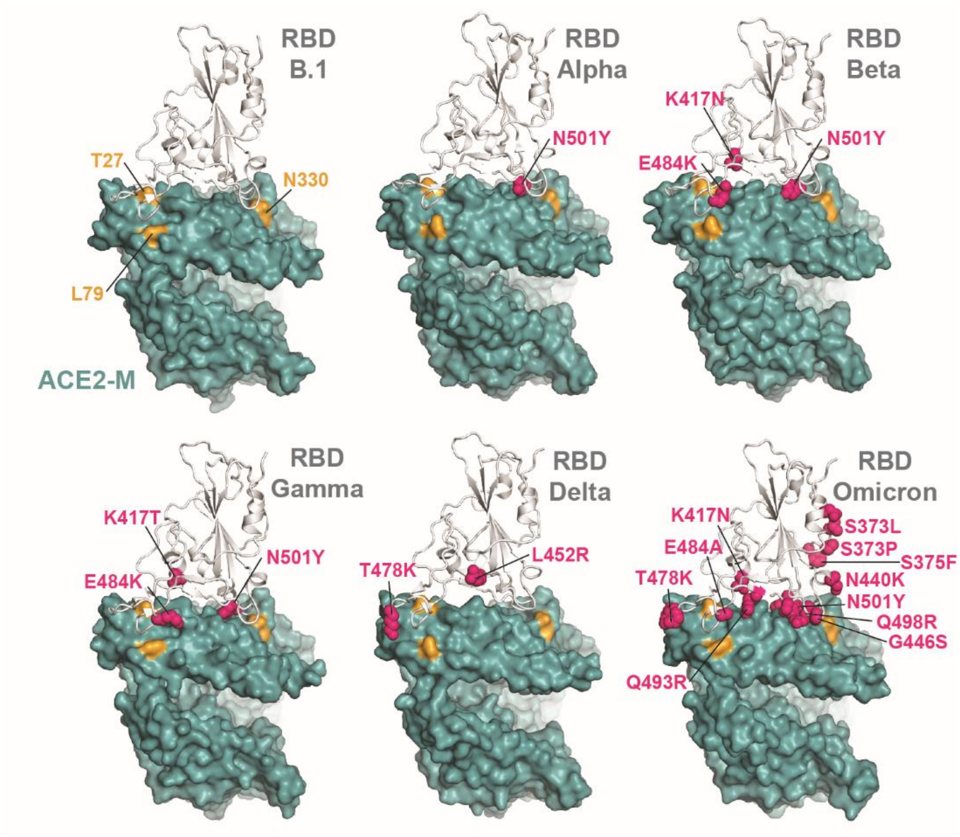

Supplementary Figure 2. Modeling of ACE2-M binding to the RBD of different variants of the spike protein.

Top left: Structure of S-RBD (grey) in complex with ACE2 (teal; PDB 6M0J). Residues substituted in ACE2-M are shown in gold. Top middle/right and bottom panels: Structure of ACE2 in complex with variant S-RBDs. RBD mutations relative to the B.1 S-protein shown in red spheres. PBD accession codes used: 7EDJ (Alpha), 7V80 (Beta), 7NXC (Gamma), 7V8A (Delta), 7VX5 (Kappa), 7T9L (Omicron).

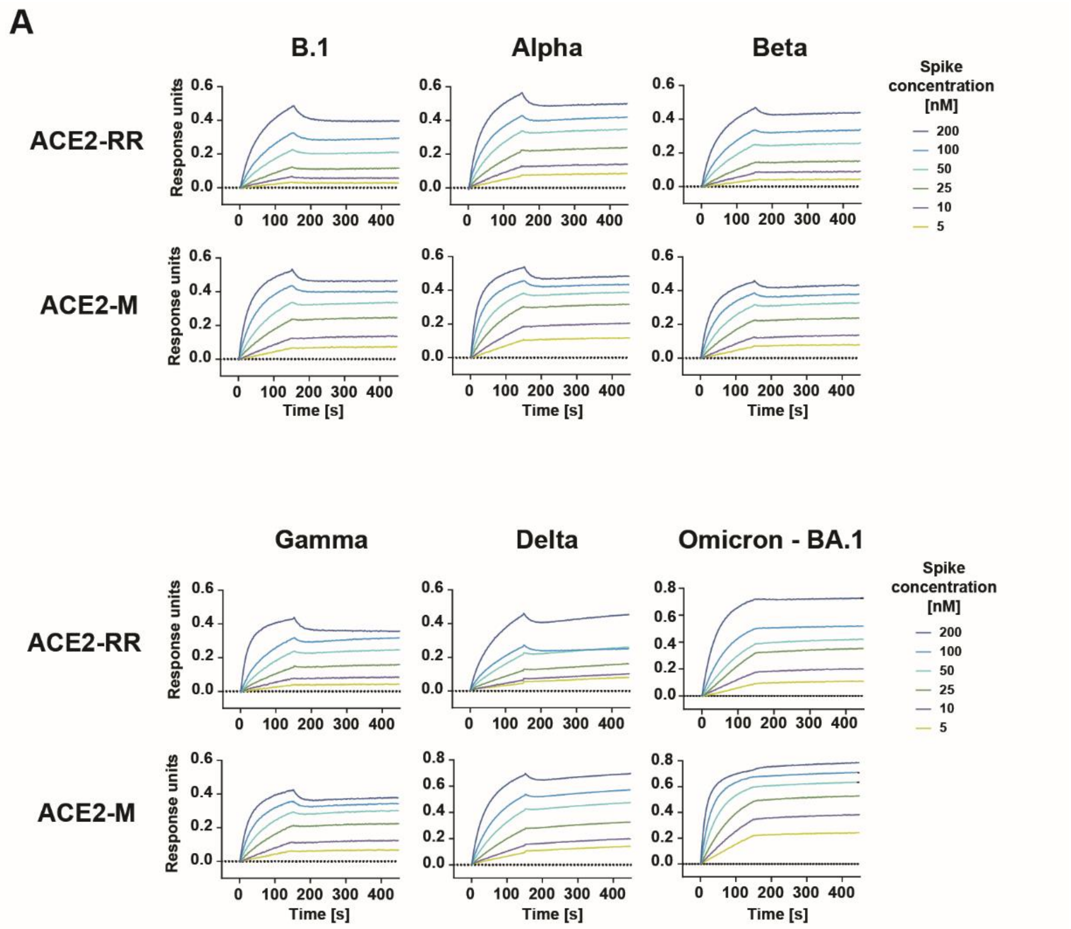

B

Construct	Trimeric Spike	K_a [1/Ms]	K_d [1/s]	K_D [nM]	X^2	R^2
ACE2-RR	B.1	7.09E+04	3.03E-03	42.7	0.08	0.99
	Alpha	1.18E+05	1.83E-03	15.5	0.32	0.99
	Beta	9.55E+04	1.06E-03	11.3	0.10	0.99
	Gamma	1.21E+05	9.88E-04	8.15	0.04	0.99
	Delta	6.23E+04	2.88E-03	46.3	0.09	0.99
	Omicron - BA.1	1.19E+05	4.75E-07	0.004	1.04	0.99
ACE2-M	B.1	1.45E+05	1.62E-03	11.1	0.23	0.99
	Alpha	2.20E+05	1.54E-03	7.01	0.48	0.99
	Beta	1.55E+05	9.10E-04	5.85	0.17	0.99
	Gamma	1.64E+05	2.04E-03	12.3	0.19	0.99
	Delta	1.25E+05	1.36E-03	10.8	0.12	0.99
	Omicron - BA.1	2.30E+05	5.26E-07	0.002	1.54	0.99

Supplementary Figure 3. Binding kinetics of ACE2-M to different S-protein variants.

(A) Trimeric full-length S protein association (t= 0 to 150s) and dissociation (t > 150 s) to immobilized ACE2-RR or ACE2-M was measured by BLI using OCTET HTX. **(B)** Table summarizing the kinetic values. R2 and X2 estimate the goodness of curve fitting. Acceptable values were considered R2 above 0.95 and X2 below 3.

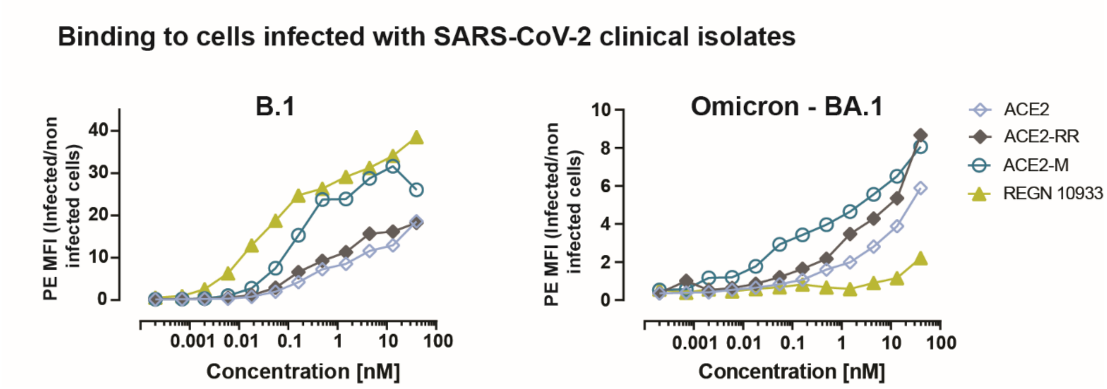

Supplementary Figure 4. Binding capacity of ACE2-M to Omicron variant

ACE2 proteins or REGN 10933 were titrated on Caco-2 cells infected with SARS-CoV-2 parental and Omicron clinical isolates. Binding was assessed by flow cytometry.

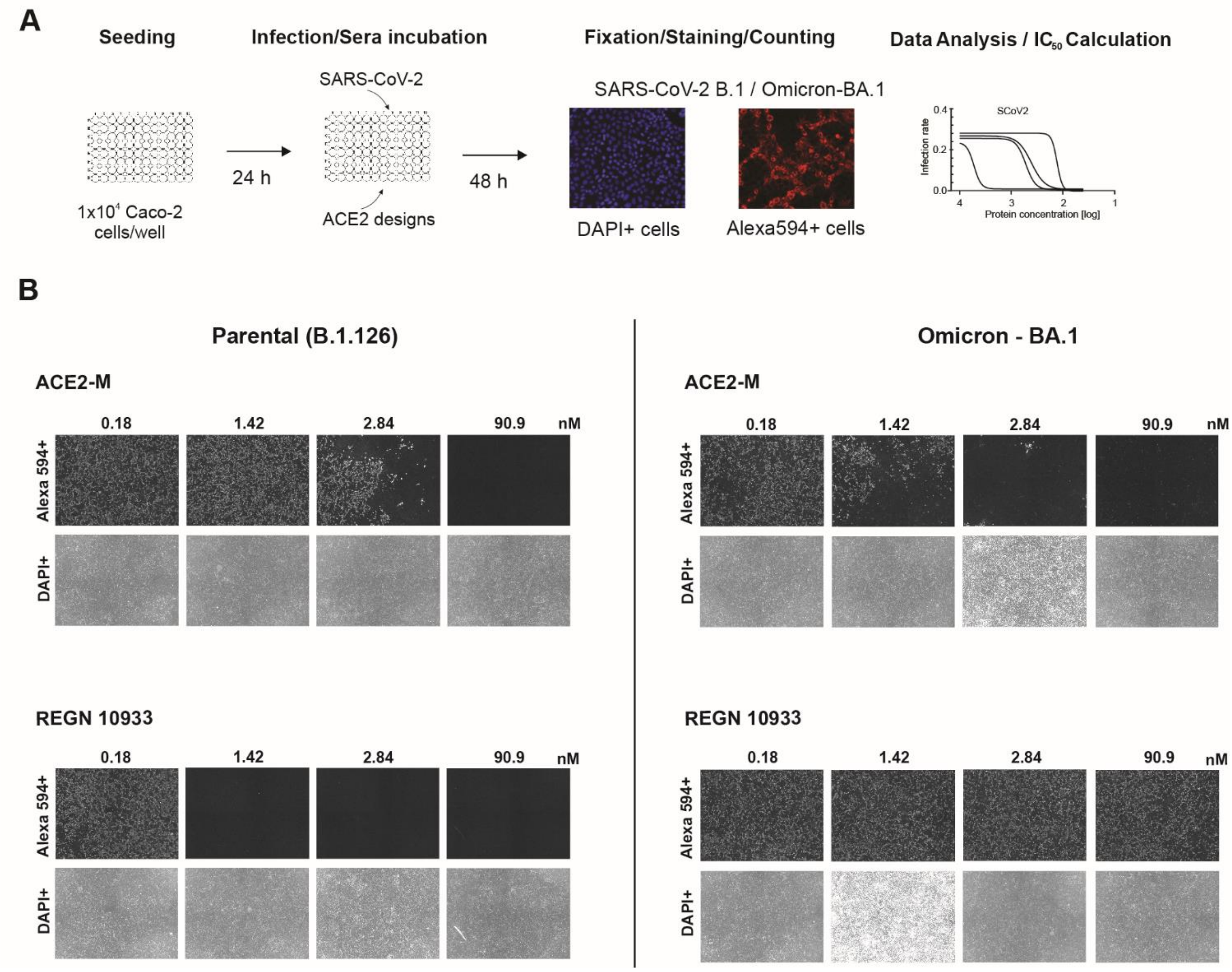

Supplementary Figure 5. Neutralization of SARS-CoV-2 clinical isolates by ACE2-protein designs vs. REGN 10933.

(A) Experimental layout of the neutralization assays employed using the two clinical isolates, SARS-CoV-2 B.1 and Omicron-BA.1 variant. **(B)** Representative fluorescence microscopy images corresponding to the neutralization assays depicted in figure 2B. Images were taken at 4-fold magnification. In the upper row of each set, infected cells are visualized and indicated as Alexa 594 positive cells. In the lower row of each set, the total amount of cells for each well is shown as DAPI-positive cells. The protein or antibody concentration (nM) is indicated above the images. The treatment and virus variant used are displayed on the right side.
